# Optimization of injections with speculum-compatible devices to deliver ethyl cellulose-ethanol into the cervix to treat cervical dysplasia

**DOI:** 10.21203/rs.3.rs-7705188/v1

**Published:** 2025-10-22

**Authors:** Taya Lee, Vené Richardson-Powell, Gatha Adhikari, Brian Crouch, Nimmi Ramanujam, Jenna L. Mueller

**Affiliations:** University of Maryland, College Park; University of Maryland, College Park; University of Maryland, College Park; Duke University; Duke University; University of Maryland, College Park

**Keywords:** Injection device, intracervical injection, ethanol ablation, ethyl cellulose, cervical dysplasia

## Abstract

Intracervical injections directly delivery therapies into the cervix. We previously explored ethyl cellulose (EC)-ethanol intracervical injections as a treatment for cervical dysplasia in low- and middle-income countries. Here we: 1) compared swine and human cervices to assess swine as a model for intracervical injections, and 2) evaluated two speculum-compatible injectors: a custom single needle injector and an extender injector, assembled from off-the-shelf components, to determine what parameters produced optimal distribution. Mechanical properties of swine and human cervices were compared. Swine cervices were injected with EC-ethanol iohexol using both injectors. Distribution and leakage volumes in tissue was visualized with microCT and quantified with 3D Slicer. Mechanical testing showed swine and human cervical tissue yielded comparable storage and loss moduli (p > 0.05). *Ex vivo* studies showed injections ≥ 10 mm deep and < 2 mL significantly reduced backflow and crack formation for both injectors. Additionally, the extender injector produced significantly less crack formation than the single needle injector. These findings indicate swine cervices are a clinically relevant model for intracervical injection studies. The extender device when inserted ≥ 10 mm and delivering < 2 mL of EC-ethanol achieved the most consistent results across intracervical delivery protocols.

## Introduction

Intracervical injections are common procedures performed by clinicians using needle and syringe to inject drugs into the cervix. Examples include injecting lidocaine prior to surgical procedures, injecting contrast agents for lymph node mapping during cancer excisions, and intratumoral injection of vaccines or other therapeutics. Lidocaine is commonly injected to provide pain relief prior to procedures such as loop electrosurgical excision procedure (LEEP), cervical biopsy, or intrauterine device placement and removal.^1–3^ Specifically, 1% lidocaine dissolved in water is injected 1–2mm deep into the ectocervix using a 5 mL syringe and 25 to 27 gauge needle.^3^ Lymph node mapping is commonly used for staging and detection of endometrial and cervical cancers that have spread into nearby nodes in the reproductive tract.^4–6^ Typically ~ 1 mL of indocyanine green (ICG) is injected at different depths ranging from 5 mm to 1 cm deep in the 3 and 9 o’ clock positions of the cervix using needle and syringe.^7^ Suspicious lymph nodes are then identified and removed by surgeons using near-infrared fluorescence imaging.^7^ Intratumoral treatment delivery is a developing field where vaccines and therapeutics are delivered directly to precancerous or cancerous lesions in the cervix. Several groups have investigated intratumoral delivery of human papillomavirus (HPV) vaccines and other cancer therapies like recombinant human adenovirus type 5 (H101) for treatment of cervical cancer in mouse models and in humans diagnosed with persistent, recurrent, or metastatic cervical cancer.^8–11^

Our group has previously investigated injecting ethanol into the cervix as a way to treat cervical dysplasia in low- and middle-income countries (LMICs).^12^ Specifically, we combine ethanol with a biocompatible polymer, ethyl cellulose (EC) to induce gel formation when injected into tissue, which leads to a controlled region of necrosis. When comparing EC-ethanol to a clinically used therapy, thermocoagulation, in a large live animal model, we found that safety endpoints were not significantly different.^13^ Additionally, one injection of 0.5 mL of EC-ethanol led to a necrotic volume that was about half that of thermocoagulation, indicating the two EC-ethanol injections could achieve a comparable zone of ablation.^13^ We also investigated what ranges of EC concentrations and injection parameters (injection depth, injection rate, injection volume) led to optimal depot formation (i.e. the volume around the needle tip) and minimized leakage away from the injection site in tissue-mimicking phantoms and excised swine cervices.^14^ Results in tissue-mimicking phantoms indicated that ≥ 6% EC-ethanol and ≥ 13 mm injection depths decreased backflow to the surface. Further, if injection depth was ≥ 13 mm, injection rate did not impact depot volume and therefore was not essential to control.^14^ Results in excised swine cervices indicated that < 3 mL injection volumes decreased leakage into cracks leading away from injection sites within the tissue.^14^

Based on these findings, we designed three speculum-compatible injectors, which include a single needle device, an extender device, and a multi-needle device. The single needle injector is a minimalistic device that includes an 8.9 cm-long needle, over-molded custom grip needle extender, and an adjustable needle depth stop.^14,15^ The extender device also has a minimalistic design but is made from commercially available components, which include a needle, needle extender, and an adjustable needle stopper.^15^ Conversely, the multi-needle device can simultaneously perform 3 injections but is more complex and bulkier. It uses air pressure from a canister to push injectate out of three equidistant needles simultaneously.^15^ In bench tests and usability studies by gynecology providers, we found the single needle and extender led to the fastest injection rates. Bench tests also showed that the single needle and extender had no ejection volume variability. Providers also preferred the ease of use, size and shape of the extender and single needle when comparing all three devices.

Based on bench and usability studies, here we further evaluated the extender and single needle devices.^15^ Specifically, we tested various injection parameters using bench tests, *ex vivo* swine cervical tissue, and *ex vivo* human cervical tissue. Results will inform which injection protocols are ideal for clinical translation of EC-ethanol for treating cervical dysplasia and provide a framework for other groups interested in optimizing intracervical injections for a variety of applications.

## Materials and Methods

### Devices

**Figure 1:**
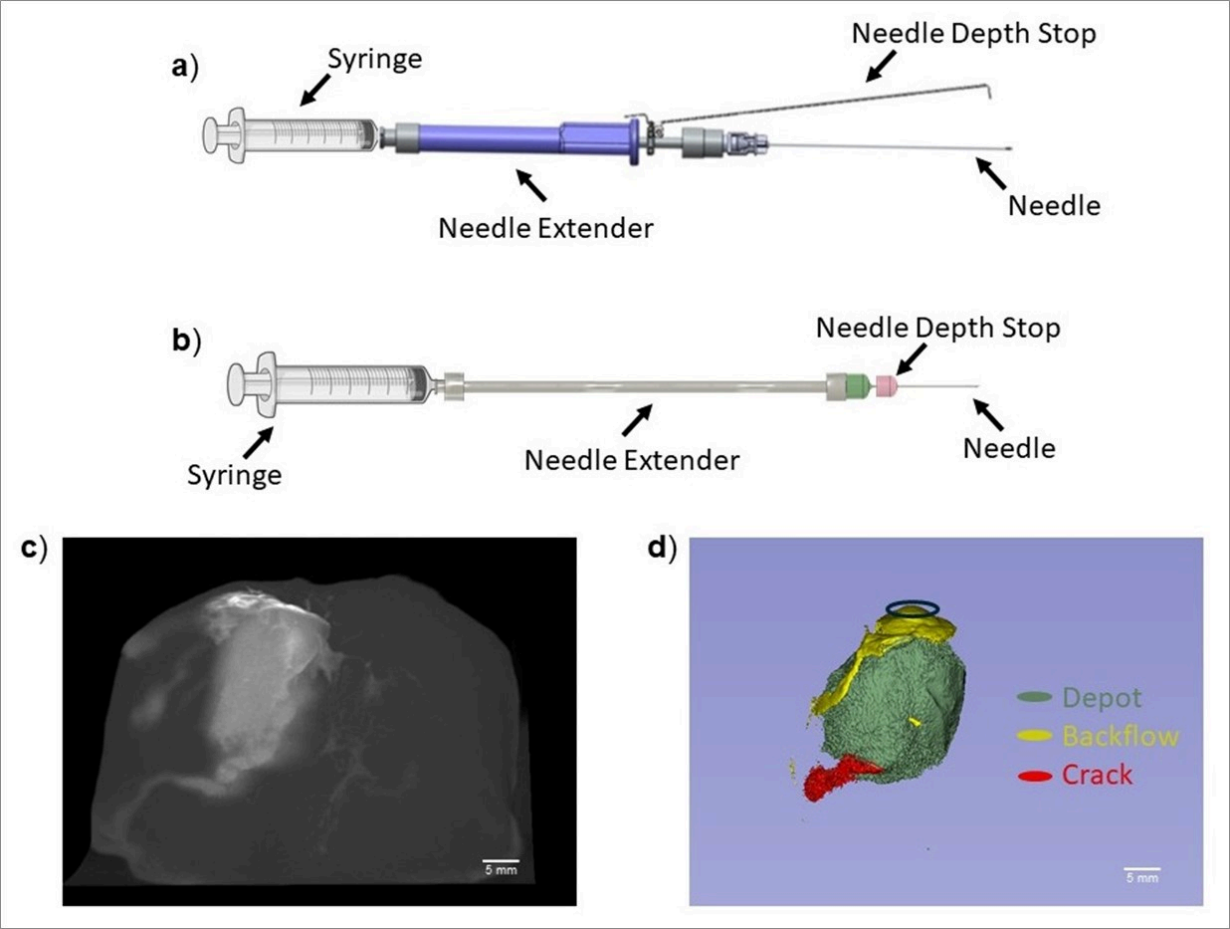
(**a**) The single needle injector device and (**b**) the extender injector device. (**c**) Reconstructed *ex vivo* swine CTVox images after EC-ethanol iohexol injections. (**d**) Corresponding 3D Slicer image highlighting: backflow of the injectate (yellow) at the needle insertion site, crack formations (red), and the depot (green). Scale bars = 5 mm.

The single needle device is a handheld speculum-compatible injector described in previous studies.^13^ Briefly, it includes a 22G 8.9 cm bevel spinal needle (4871835, Becton, Dickinson and Company, Franklin Lakes, NJ), custom grip over-molded needle extender, an adjustable needle depth stop, and a 3 mL syringe (309657, Becton, Dickinson and Company, Franklin Lakes, NJ) (Table 1 and Fig. 1a). The extender device includes several commercially available components and has been previously described. Briefly, the device includes a 22G 2.54 cm bevel needle (305155, Becton, Dickinson and Company, Franklin Lakes, NJ), a 15.2 cm reusable needle extender (965196, Sklar, West Chester, PA), and a 3 mL syringe (Table 1 and Fig. 1b).

#### EC-ethanol iohexol preparation:

Solutions of EC-ethanol were prepared by mixing 200 proof ethanol (Koptec, King of Prussia, PA, USA) and ethyl cellulose (Sigma Aldrich, St. Louis, MO, USA) to a concentration of 6% (EC:ethanol, w:v). Then iohexol (Omnipaque, GE Healthcare, Chicago IL) was added to the EC-ethanol solution at a concentration of 40 mg/mL, which optimized visualization of gel depots in cervical tissue.^14^

### Procedure setup

Both devices were used in a vertical orientation to manually inject EC-ethanol into various samples. Each device was secured in a vise tool (PanaVise, Reno, NV, USA) positioned on top of a lab lift (Eisco, Rochester, New York). Each device was first primed with pure 200 proof ethanol before being connected to a 3 mL syringe containing 6% EC-ethanol iohexol. The loaded syringe plunger was pushed until there was a flow of gel from the tip of the needle. The syringe was then reloaded with 6% EC-ethanol iohexol prior to experiments.

### Bench Test

An overview of the parameters used in each experiment is summarized in Table 1. First, ejection volumes and rates were determined by ejecting various volumes (0.5, 1, 2, and 3 mL) of 6% EC-ethanol iohexol into microcentrifuge tubes using both devices. Thirty-six microcentrifuge tubes were collected (n = 18 for each device, n = 6 for each volume group). During each experiment, the time to complete the ejection was recorded. After the ejection was completed, the device remained in place for five minutes, and the final volume in the microcentrifuge tube was recorded. The ejection rate was calculated by dividing the final volume by the ejection time.

### Tissue preparation

To determine the distribution volume of EC-ethanol by each device, both excised porcine and human cervical tissue were used. The porcine gynecological tissue was purchased from Animal Biotech Industries (Doylestown, PA, USA). The vaginal canal and the uterus were removed from each sample and the cervix was sectioned into 2.5 cm segments. Each section was placed into 10% PBS until injections were performed. This protocol was approved by the University of Maryland, College Park Institutional Animal Care and Use Committee (IACUC, Protocol # R-AUG-23–40). De-identified human cervix tissue samples were collected post autopsy by University of Maryland School of Medicine (Baltimore, MD, USA) pathologists. All experiment protocols of this study were approved by the University of Maryland’s Institutional Review Board and designated ‘Not Human Subjects Research’ (Protocol #HP-00081117). All *ex vivo* tissue experiments were performed in accordance with relevant guidelines and regulations. All methods are reported in accordance with ARRIVE guidelines. The samples were stored on ice in 10% PBS and received for experiments within two hours of collection. Once the samples were procured, they were placed into a larger container of 10% PBS to prepare for injections and/or mechanical testing. All experiments were performed within 24 hours of receiving tissue samples. Cervix samples were dissected to remove excess fat and residual tissue. For rheometric testing, the cervix was sectioned laterally to obtain samples that fit the 25 mm circular plate of the AR-G2 rheometer (TA Instruments, New Castle, DE).

### Modulus testing

The modulus testing for the excised swine and human cervical tissue was performed using an ARES-G2 rheometer machine (TA instruments, New Castle, DE, USA) to compare their mechanical properties and evaluate feasibility of swine tissue as model for human injections. The ARES-G2 was equipped with a 25 mm circular steel parallel top plate and standard Peltier bottom plate. Mechanical testing followed methods previously described.^12^ Briefly, before testing residual fat was removed, and each section was placed in an incubator at 37°C for 1 hour prior to experiments. 150 grit sandpaper was attached to both the top plate and bottom plate to prevent tissue sliding during compression. Frequency sweeps were conducted from 0.1 to 10 rad/s within the linear viscoelastic region at a constant strain amplitude of 5% to obtain storage moduli (G’) and loss moduli (G”).

### Ex vivo injection procedure

In preparation for all *ex vivo* experiments, needle length was adjusted and confirmed using digital calipers before the devices were secured in the vertical orientation. For depth comparison experiments, needle length was adjusted between 6 to 13 mm. Here shallower depths < 13 mm was tested in tissue as tissue creates more of a seal with the needle interface (compared to tissue-mimicking phantoms, which tear easily). The needle length was set to 13 mm for volume comparison experiments. After following the procedure setup methods, the ectocervix of each cervical section was placed below the device with the cervical os facing upward. The needle was then manually inserted halfway between the cervical os and the outer edge of the cervix with the bevel of the needle facing outward from the endocervical canal. The needle was inserted until the needle depth stop was flush with the tissue. While performing the injection, the injection time was recorded. After each injection, the devices were left in place for 5 minutes to reduce backflow.

### Imaging and image analysis

Imaging and image analysis was done using previously described techniques.^14–16^ Briefly, the excised tissue was imaged with a Bruker micro-CT machine (Bruker SkyScan 1276, Billerica, MA). Step-and-shoot projections were taken with a 9.31 × 158.75 cm field of view and 360° rotation (70 kV, 200 μA, 50 ms exposure time, 0.5 mm aluminum filter). Projections were then reconstructed using NRecon (Bruker) and CT Vox (Bruker) software used to create and edit 3D images. Each image was edited using post-alignment correction, ring correction, and beam-hardening correction.

EC-ethanol iohexol distributions were then quantified using 3D Slicer software (Kitware, Clifton Park, NY). This was done by applying Renyi entropy thresholding and manually adjusting the thresholding values to segment EC-ethanol within the tissue and remove noise. The depot was identified as the main ellipsoidal region that formed around the needle (Fig. 1d). Ideally, depot volume is equivalent to total distribution volume and fully covers the diseased tissue region. However, injections can also result in the formation of cracks into which the injectate flows or backflow to the tissue surface. Here crack formation was identified as long trails of injectate that travel away from the main depot within the tissue (Fig. 1d). This phenomenon can cause off target necrosis in healthy tissue. Backflow formation is identified as the retrograde flow of the 6% EC-ethanol injectate to the tissue surface (Fig. 1d). This can increase the amount of EC ethanol necessary for complete ablation and cause off target necrosis on the surface of the cervix. Thus, additional segmentations are created to individually quantify crack formation and backflow. Specifically, once EC-ethanol was segmented, the total distribution volume was calculated and then further segmented into three parameters: 1) depot volume, 2) crack formation, and 3) backflow and labeled as such. The segmentations for each threshold were manipulated by manually cutting away sections of the EC-ethanol distribution not representative of that segment (Fig. 1d). The segmentations were then quantified to calculate the volume distribution for each parameter. The ratio for each parameter was calculated by dividing the parameter of interest by the initial volume.

### Statistical analysis

A sample size of n = 5–6 trials were performed for each parameter tested. Kruskal Wallis one-sided parametric ANOVA tests with Dunn’s test p-value corrections or Wilcoxon rank sum tests were performed in MATLAB (MathWorks, Natick, MA, USA) to assess significant differences in bench test and tissue experiments. Rejection of the null hypothesis was determined by significance levels of p < 0.05.

## Results

### Excised human and swine cervix tissue yield similar circular EC-ethanol depots and mechanical properties

**Figure 2:**
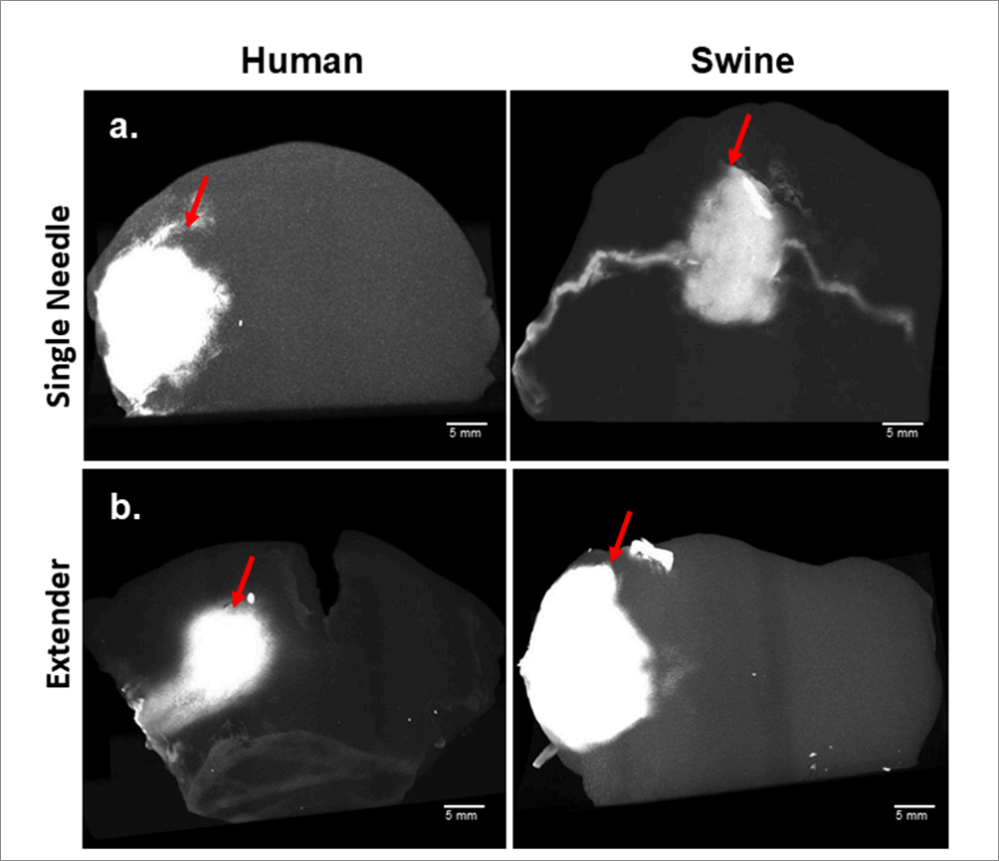
Side view of reconstructed *ex vivo* human (left) and swine tissue (right) imaged by the CTVox after injecting 6% EC-ethanol-iohexol with (**a**) the single needle injector device and (**b**) the extender device injector device. Each injector device uses a 22G needle, was inserted at 13mm, and was injected with 1 mL. Red arrows indicate injection site. Scale bars = 5 mm.

Excised human and swine cervix tissue were both injected with 6% EC-ethanol-iohexol to evaluate the shape of EC-ethanol depots. All tissue was injected with the extender and single needle devices using 22G needles, 13 mm injection depths, and 1 mL injection volumes. After microCT imaging and reconstruction, we found both devices created similar circular EC-ethanol deposits in human vs. swine tissue (Fig. 2).

**Figure 3:**
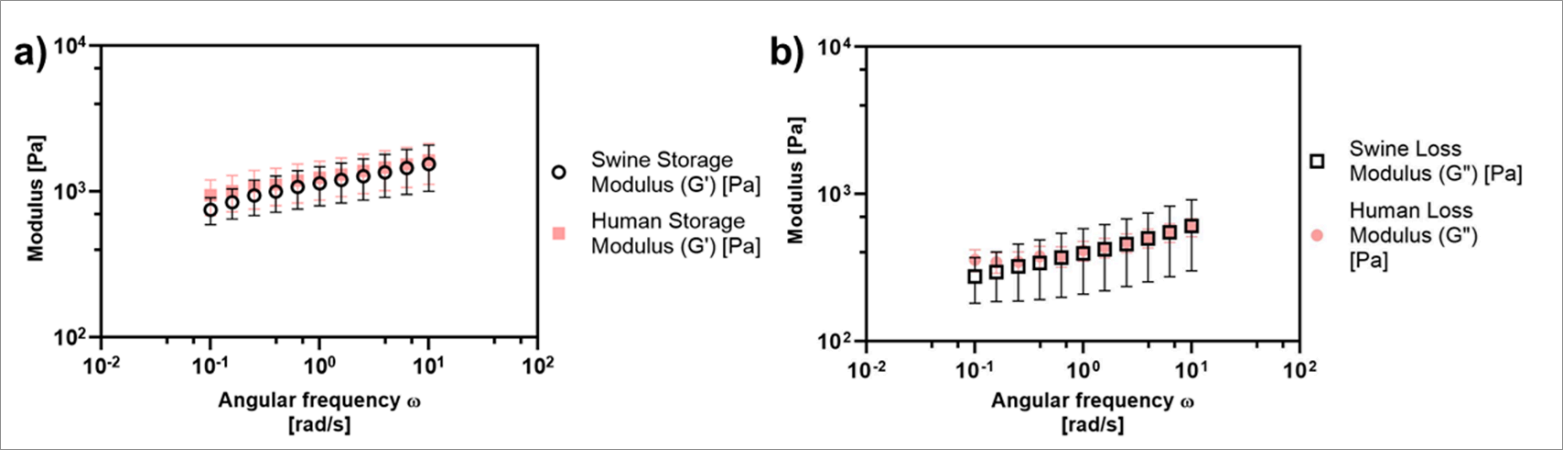
Mechanical testing of *ex vivo* human and swine tissue comparing (**a**) storage modulus and (**b**) loss modulus. Sample size n = 6.

To ensure that the mechanical properties of the human and swine tissue were similar, we characterized the storage and loss moduli of tissue samples using a rheometer (Fig. 3). Rheological characterization revealed that both human and swine cervical tissues exhibit viscoelastic behavior characterized by frequency-dependent increases in both storage (G′) and loss (G″) moduli. Across the physiological frequency range (0.03–10 rad/s), the storage modulus (G′), which reflects elastic stiffness, increased modestly by ~ 1.5-fold in both tissues, ranging from approximately 800 to 1300 Pa (Fig. 3a). The loss modulus (G″), which represents viscous dissipation, showed a more pronounced ~ 2.5–3-fold increase, ranging from ~ 160 to 520 Pa (Fig. 3b). At every frequency tested, swine tissue exhibited slightly higher G′ and G″ values than human tissue (typically within 10–25%), although the differences were not statistically significant **(p > 0.05)**. These results indicate that both tissues are predominantly elastic (G′ >G″ across all ω) and share remarkably similar viscoelastic profiles, suggesting swine cervices are an appropriate model for tissue testing.

### Bench testing indicates extender and single needle devices produced similar ejection volumes but different ejection rates

**Figure 4:**
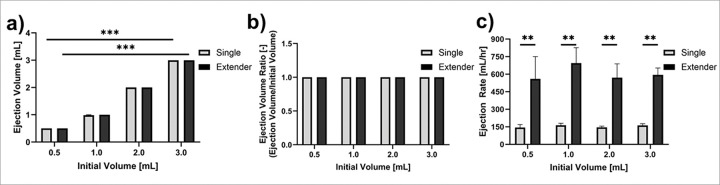
Benchtop experiments comparing different initial volumes. (**a**) Total ejection volume, (**b**) ejection volume ratio, and (**c**) ejection rate for volumes 0.5, 1, 2, and 3 mL performed with the single needle device and the extender device. Error bars are standard error (*p < 0.05, **p < 0.005, ***p < 0.0005).

Ejections with both devices were performed into graduated microcentrifuge tubes to evaluate differences in ejection volume and rate. No significant differences in ejection volume and ejection volume ratios were observed when comparing the two devices at the same initial volumes (Fig. 4a–b). However, a significant increase in ejection volume was observed for 0.5 mL vs. 3 mL for both the single needle **(p = 0.00012)** and extender **(p = 0.00012)** (Fig. 4a). When comparing ejection rates, the extender device ejections were significantly faster **(p < 0.005)** (Fig. 4c). The extender produced ejection rates of 605.02 mL/hr ± 136.11 on average while the single needle injector averaged ejection rates of 154.22 mL/hr ± 19.10 (Fig. 4c).

### Testing in swine cervices indicates injections deeper than 8 mm with volumes less than 2 mL led to less backflow and crack formation for both devices

**Figure 5:**
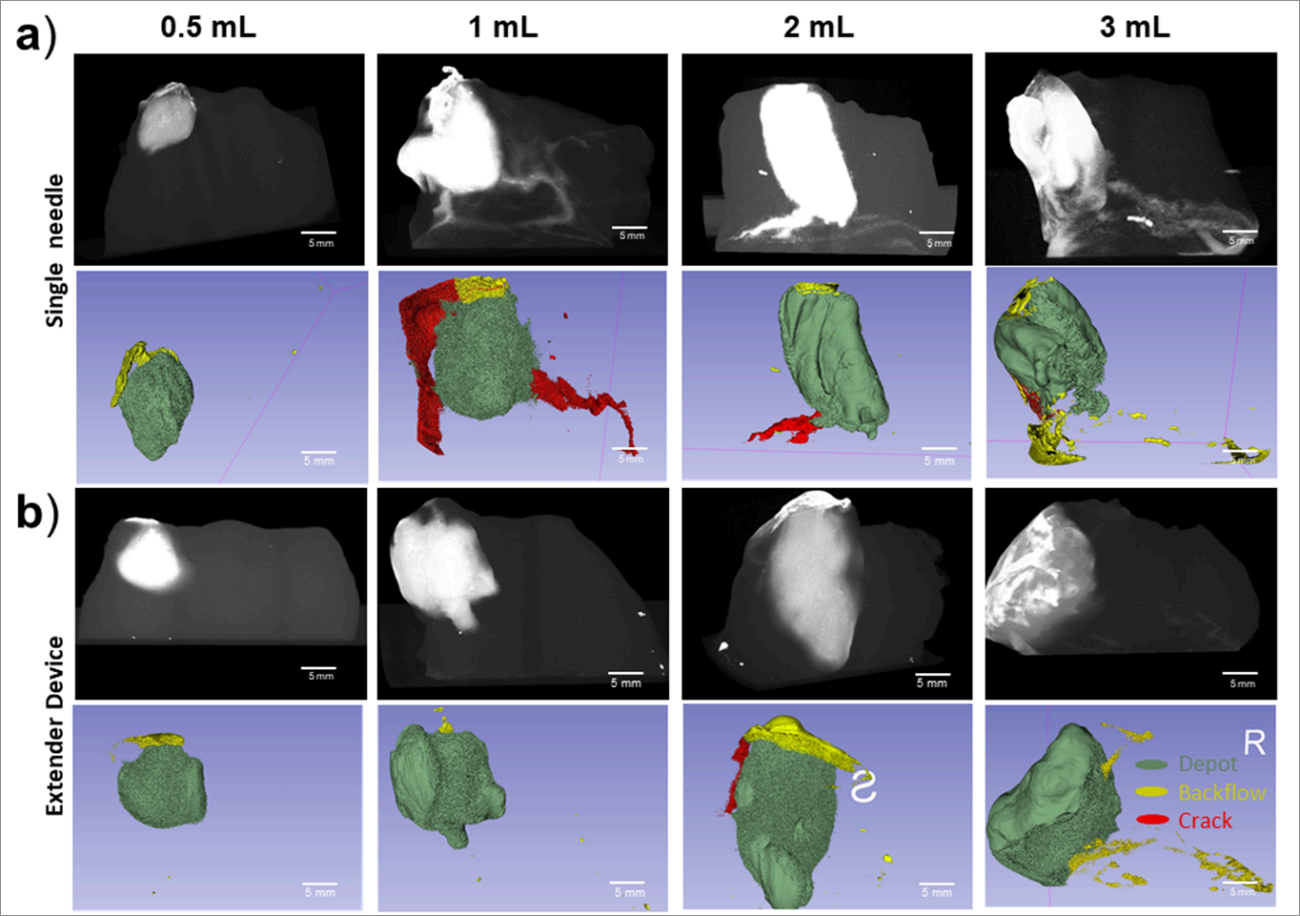
Distribution volume comparison between devices in *ex vivo* swine cervical tissue. Representative reconstructed post injection CT images and 3D Slicer images of depots are shown in rows 1 and 2, respectively for the (**a**) single needle and (**b**) extender injection devices. Images represent initial injection volumes of 0.5, 1, 2, and 3 mL performed with each device. Scale bars = 5 mm.

Injections with both devices were tested in swine cervices to evaluate the range of injection volumes (0.5, 1, 2, and 3 mL) and injection depths (6, 8, 10, and 13 mm) that led to good depot formation with minimal leakage. Figure 5 shows that good depot formation was achieved with both devices across the range of volumes that were tested. However, more crack formation was observed with the single needle device, particularly for volumes larger than 0.5 mL, and slightly more backflow was observed for injection volumes ≥ 2 mL.

**Figure 6:**
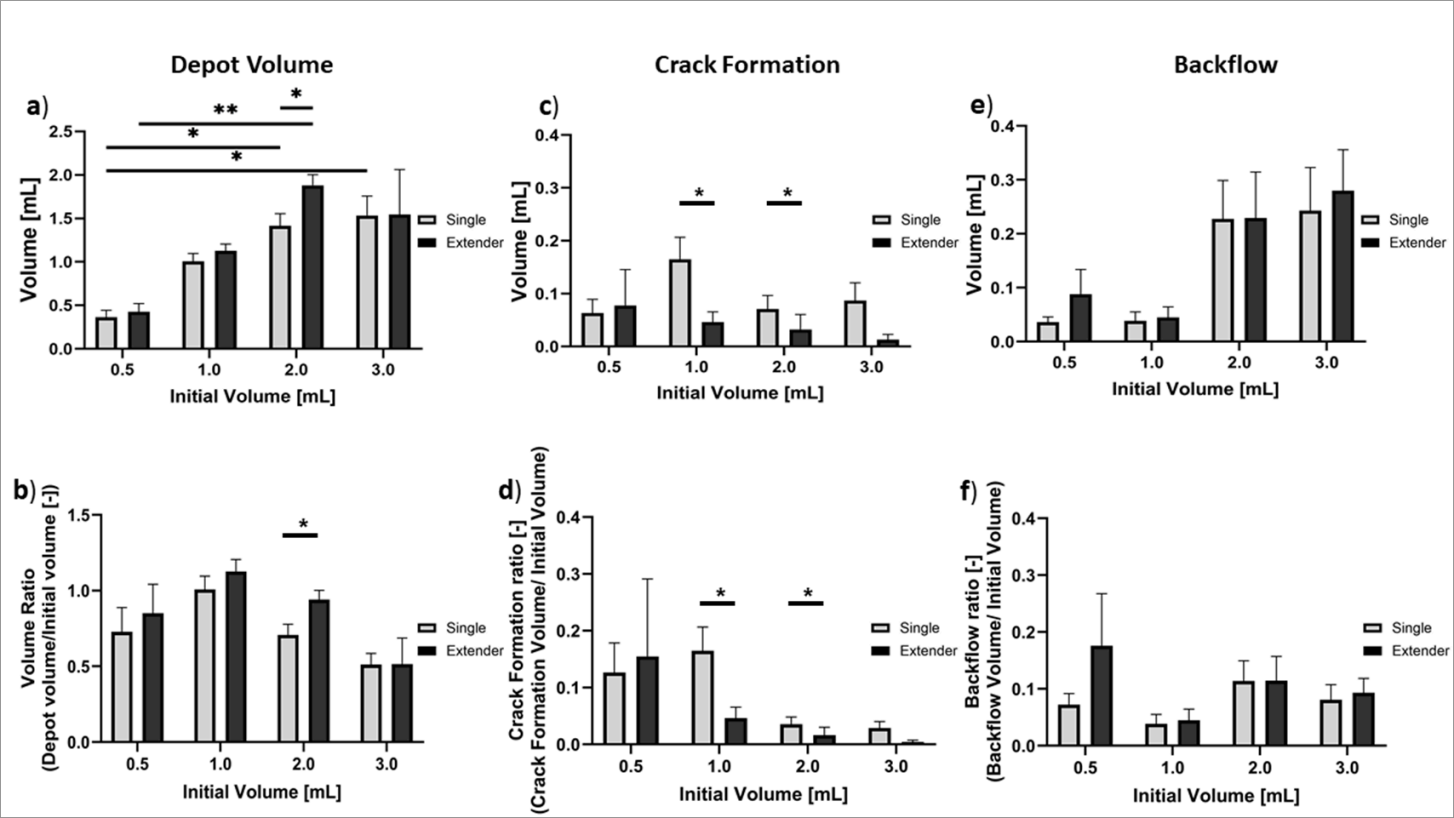
(**a**) Depot volume and (**b**) volume ratio achieved with the single needle and extender devices with different initial volumes. Quantification of EC-ethanol-iohexol that leaked from target site including: (**c**) crack formation volume, (**d**) crack formation ratio, (**e**) backflow volume, and (**f**) backflow ratio. Error bars are standard error (n = 6 per group, *p < 0.05, **p < 0.005, ***p < 0.0005).

The depot, crack formation, and backflow volumes and ratios were calculated for each injection. The extender needle device led to slightly higher depot volumes and volume ratios when compared to single needle device for all volumes with significantly higher depot volumes for 2 mL injections **(p = 0.041**, Fig. 6a–b). For injections with the single needle device, depot volume was significantly higher for 0.5 vs. 2 mL **(p = 0.044)** and 0.5 vs. 3 mL **(p = 0.022)** injections. For the extender device injections, depot volume was significantly higher for 0.5 vs. 2 mL **(p = 0.001)**. The single needle device led to significantly more crack formation volume than the extender needle device for 1 mL **(p = 0.026)** and 3 mL **(p = 0.020)** volumes (Fig. 6c–d) likely due to the increased pressure build-up within the single needle device. Lastly, 1 mL injections led to the lowest backflow volumes and ratios while 2 and 3 mL injections led to more backflow for both devices though differences were not significant (Fig. 6e–f). Taken together, 1 mL injections with the extender needle device led to the highest depot volume ratio and had minimal crack formation and backflow volume.

**Figure 7:**
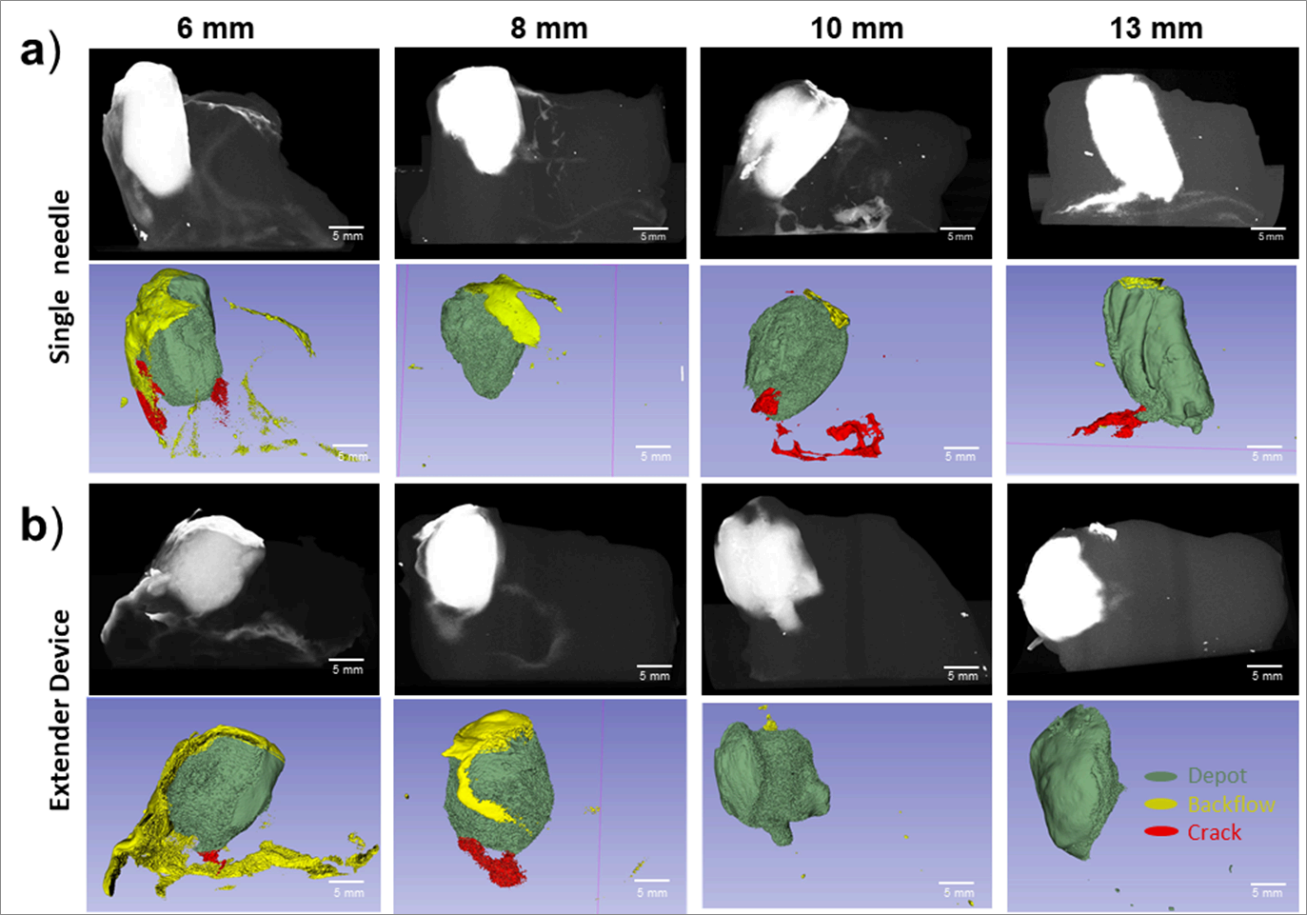
Depth comparison between devices in *ex vivo* swine cervical tissue. Representative reconstructed post injection CT images and 3D Slicer images of depots are shown in rows 1 and 2, respectively for the (**a**) single needle and (**b**) extender injection devices. Images represent injections performed with each device at depths of 6, 8, 10, and 13 mm. Scale bars = 5 mm.

Figure 7. Depth comparison between devices in *ex vivo* swine cervical tissue. Representative reconstructed post injection CT images and 3D Slicer images of depots are shown in rows 1 and 2, respectively for the (**a**) single needle and (**b**) extender injection devices. Images represent injections performed with each device at depths of 6, 8, 10, and 13 mm. Scale bars = 5 mm.

**Figure 8:**
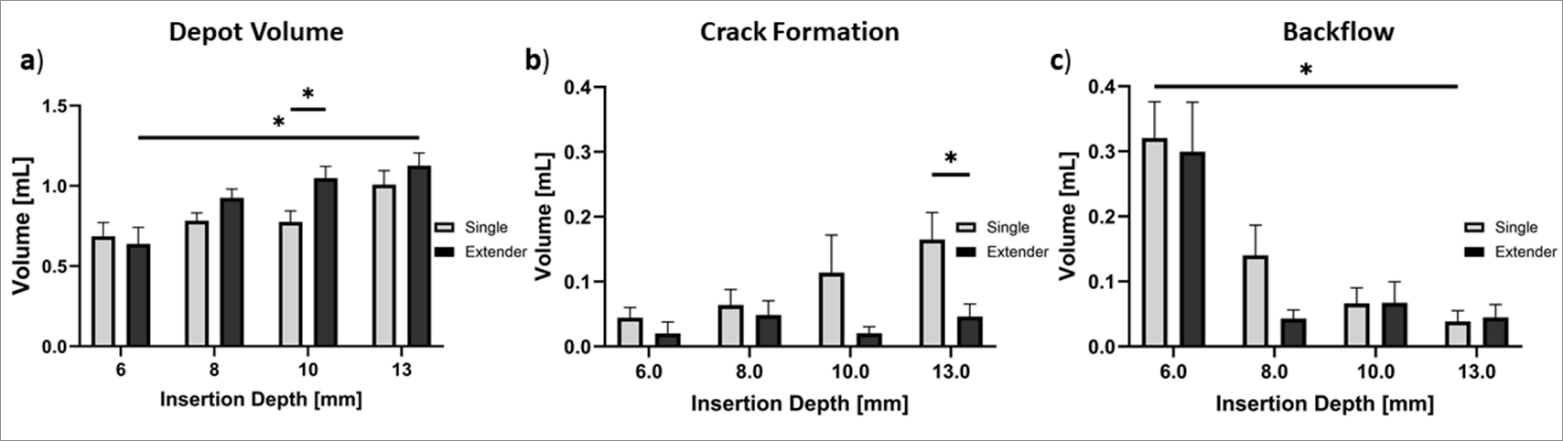
(**a**) Depot volume achieved with the single needle and extender devices with different needle insertion depths. Quantification of EC-ethanol-iohexol that leaked from target site including: (**b**) crack formation volume and (**c**) backflow volume. Error bars are standard error (n = 6 per group, *p < 0.05, **p < 0.005, ***p < 0.0005).

Figure 8a shows that depot volumes increased as insertion depths increased for both devices. 13 mm insertion depths led to the greatest depot volumes for both devices. Specifically, 13 mm injections with the extender needle device led to significantly higher depot volumes than 6 mm injections **(p = 0.025)**. The single needle device led to higher crack formation volumes compared to the extender device for all injection depths (Fig. 8b) with significant differences observed at 13 mm **(p = 0.026)**. The highest backflow volume was observed at 6 mm depths for both devices (Fig. 8c). More specifically, 6 mm single needle device injections led to significantly higher backflow volume and backflow volume ratios **(p = 0.017)** when compared to 13 mm injection depths (Fig. 8c). Taken together, 10 and 13 mm deep injections with the extender needle device led to the highest depot volume ratio and had minimal crack formation and backflow volume.

## Discussion

Intracervical injections involve directly injecting drugs into the cervix; common examples include injecting lidocaine prior to surgical procedures, injecting contrast agents like ICG for lymph node mapping during cancer excisions, and intratumoral injection of vaccines.^1,2,5,8,9^ While intracervical injections are common, the influence of injection parameters on drug distribution in the cervix has not been thoroughly studied. Here we investigated what injection parameters (and what ranges of those injection parameters) matter most when injecting phase-transitioning therapeutics, which is a growing area of medical research.^17–20^ Specifically, we selected ethanol as the therapeutic and EC as the component that forms a gel when injected into tissue. However, the insight and methodology described here can be extended to other drugs and hydrogel delivery systems. Because the cervix is approximately 7–13 cm inside in the vaginal canal, a speculum-compatible injector must be carefully designed to reach the cervix while enabling the provider to simultaneously visualize the cervix and control any important injection parameters. Previously, we designed speculum-compatible needle injectors that were evaluated by OB-GYNs. Two of our designs were well received by providers: 1) a single needle device, which was designed around a 22G 8.9 cm-long needle (commonly used to inject ICG into the cervix) with a footie needle depth stop to control injection depth, and 2) the extender needle device, which has a 22G 1.54 cm-long needle (the shortest 22G needle that is commercially available) with a needle depth stop to control injection depth connected to a long needle extender in order to reach the cervix. Building on prior study, here we conducted thorough evaluations with both devices on the bench and in *ex vivo* tissue (Table 1). The feasibility of *ex vivo* swine cervical tissue as a model of human cervical tissue injections were also evaluated through mechanical testing and injections.

Many researchers interested in cervical tissue use a swine model since the swine cervix is nearly human size, and humans and pigs have similar anatomical characteristics, mucosal folds, and transitions from squamous to columnar epithelium.^21^ However, to our knowledge there are no studies directly comparing the mechanical properties of excised swine and human cervical tissue. Thus, we measured the storage (G’) and loss moduli (G’’) of swine and human tissue to evaluate the elastic and viscous contributions, respectively – both of which can impact the drug distribution of phase-transitioning therapeutics like EC-ethanol. Mechanical testing showed both human and swine excised cervix tissue yield comparable mechanical properties (Fig. 3). Our results showed an average G’ of 1267 Pa and G’’ of 433 Pa for the human cervix samples. Swine cervix samples had similar results showing an average G’ of 1145 Pa and G’’ of 411 Pa. The comparable viscoelastic profiles observed between swine and human cervical tissues demonstrate the suitability of swine as a surrogate for human tissue models. Although swine tissue exhibited slightly higher storage and loss moduli across the frequency spectrum, these differences were within the margin of variability and unlikely to significantly impact applications that rely on relative mechanical performance. The dominant elastic behavior (G′ >G″) in both species suggests that tissue stiffness, rather than viscous dissipation, governs mechanical response under oscillatory loading. These findings support the use of swine tissue in translational research and device evaluation, particularly where human tissue availability is limited. To confirm that similar mechanical properties led to morphologically similar injections, we performed EC-ethanol injections in human and swine cervical tissue as seen in Fig. 2. EC-ethanol injections yielded similar depots in human vs swine tissue both in terms of size and shape. Taken together, results indicate that swine cervix tissue is a clinically relevant model for studying drug distribution resulting from intracervical injections.

To assess the performance of the single needle and extender devices, we first performed ejections into microcentrifuge tubes. This allowed us to confirm that accurate volumes were ejected from each device in a controlled environment. Bench testing indicated that the extender and single needle devices produced statistically similar ejection volumes (Fig. 4a). However, Fig. 4b shows that the single needle device led to decreased injection rates (62.5 ± 4.7 mL/hr) as volume increased, while the extender achieved statistically faster injection rates (96.4 ± 2.5 mL/hr) at volumes greater than 1 mL. This difference is attributed to differences in needle length, with the single needle device having a longer needle than the extender needle device (8.9 cm vs 2.54 cm). The needle length leads to increased pressure build up in the system thus leading to decreased injection rates.^13^

We next performed injections into *ex vivo* swine cervical tissue to study what injection parameters gave rise to fluid leakage.^22^ First, both devices were used to inject a range of initial volumes and the resulting depot, crack formation, and backflow volumes were quantified. Ideally, lesions could be fully covered with a single injection; however, previous studies have shown that as volume increases leakage increases.^14^ Thus, we sought to understand what the upper limit of a single injection should be with both devices. We also quantified crack formation leakage and backflow leakage, which can lead to off target tissue necrosis. Therefore, increasing depot volumes to cover precancerous lesions while also decreasing leakage is vital for treatment efficacy. When testing various injection volumes in excised tissue, we found that both devices achieved ellipsoidal depot volume (Fig. 5). However, higher volumes ≥ 2 mL led to increased leakage for both devices (Fig. 5, 6 and **Supplementary Fig. 1**). More specifically, both devices had decreased depot volume ratios and increased backflow for injections above 2 mLs (Fig. 6b and 6e). Therefore, for optimal injections, volumes should stay under 2 mLs. Additionally, the extender needle device had moderately higher depot volume ratios and significantly lower crack formation ratios than the single needle device (Fig. 6a–d).

Next, different needle depths were used with both devices to determine what depths led to more or less fluid leakage. Cervical dysplasia is classified into three grades based on the depth of precancerous lesions in the cervix, low grade, moderate grade, and high grade.^23^ Of the three grades, high grade dysplasia is the deepest with lesions covering the entire thickness of the epithelium (up to 5 mm deep). Other clinically used therapies that surgically excise high-grade lesions, such as LEEP and cold knife conization, typically remove 13 mm deep cone shaped resections and lead to a 95% chance of complete ablation.^24,25^ By varying insertion depth, we aimed to determine if the devices were able to treat a range of depths (6 to 13 mm) to adequately ablate high grade cervical dysplasia while reducing leakage.^23^ The extender device’s depot volume and volume ratio significantly increased as insertion depth increased from 6 to 13 mm **(p = 0.025**, Fig. 8a). The single needle injections led to higher crack formation volumes and ratios than the extender needle device at all depths (Fig. 8b). Both devices had the most amount of backflow at 6 mm and least amount of backflow at 13 mm, indicating deeper injections lead to higher injectate retention (Fig. 8c). As injection depth increased from 6 to 10 mm, backflow decreased by a factor of 4X for both the single and extender devices. Thus, insertion depths of ≥ 10 mm are recommended for both devices. Previous studies performed in 1% agar phantoms found that injections less than 13mm in depth led to significant decreases in injection volume.^13^ This depth discrepancy can be attributed to differences in compressive stress between tissue and phantoms. The higher compressive stress of tissue creates a “seal” around the needle allowing shallower depths to be performed without significant leakage.

Here, we have presented a methodology for testing intracervical injection parameters and devices. Swine and human *ex vivo* cervical tissue had similar rheology properties and depot shapes, which provided rationale for using swine *ex vivo* cervical tissue a model for human intracervical injections. We also compared two speculum compatible devices and found the extender injector device led to significantly less crack formulation volume compared to the single needle injector. Additionally, insertion depths ≥ 10 mm and < 2 mL led to optimal depot volume ratios while minimizing backflow and crack formation volumes. These results will inform future clinical trials in which the extender needle device will be used to inject EC-ethanol into the cervix of patients scheduled for hysterectomy.

## Supplementary Material

Supplementary Files

This is a list of supplementary files associated with this preprint. Click to download.

• SupplementaryFiguresoptimizationSR.docx

## Figures and Tables

**Table 1.** Overview of parameters used for each experiment.

Exp	Sample	Injector Device	Needle Gauge, Type Length	Needle Depth (mm)	Injection Volume (mL)
1	Micro-centrifuge tubes (n = 6)	Single Needle injector	22 G; bevel; 8.9 cm	N/A	0.5, 1, 2, 3
2	Micro-centrifuge tubes (n = 6)	Extender injector device	22 G: bevel; 2.54 cm	N/A	0.5, 1, 2, 3
3	Cervical Swine Tissue (n = 5℃6)	Single Needle injector	22 G, bevel; 8.9 cm	6, 8, 10, 13	0.5, 1, 2, 3
4	Cervical Swine Tissue (n = 5–6)	Extender injector device	22 G; bevel; 2,54 cm	6, 8, 10, 13	0.5, 1, 2, 3
5	Cervical Human Tissue (n = 1)	Single Needle injector	22 G; bevel; 8.9 cm	13	1
6	Cervical Human Tissue (n = 1)	Extender injector device	22 G; bevel; 2.54 cm	13	1

## Data Availability

The datasets used and/or analyzed during the current study are available from the corresponding author on reasonable request.
